# Public Perception and Reception of Robotic Applications in Public Health Emergencies Based on a Questionnaire Survey Conducted during COVID-19

**DOI:** 10.3390/ijerph182010908

**Published:** 2021-10-17

**Authors:** Hui Jiang, Lin Cheng

**Affiliations:** 1 School of Foreign Languages, Sun Yat-sen University, Guangzhou 510275, China; jianghui6@mail.sysu.edu.cn; 2 Department of German Studies, Institute of Hermeneutics, Guangdong University of Foreign Studies, Guangzhou 510420, China

**Keywords:** anti-pandemic robots, COVID-19, China, public awareness, replacement of humans by robots, roboethics

## Abstract

Various intelligent technologies have been applied during COVID-19, which has become a worldwide public health emergency and brought significant challenges to the medical systems around the world. Notably, the application of robots has played a role in hospitals, quarantine facilities and public spaces and has attracted much attention from the media and the public. This study is based on a questionnaire survey on the perception and reception of robots used for medical care in the pandemic among the Chinese population. A total of 1667 people participated in the survey, 93.6% of respondents were pursuing or had completed a bachelor, master or even doctorate degree. The results show that Chinese people generally held positive attitudes towards “anti-pandemic robots” and affirmed their contribution to reducing the burden of medical care and virus transmission. A few respondents were concerned about the issues of robots replacing humans and it was apparent that their ethical views on robots were not completely consistent across their demographics (e.g., age, industry). Nevertheless, most respondents tended to be optimistic about robot applications and dialectical about the ethical issues involved. This is related to the prominent role robots played during the pandemic, the Chinese public’s expectations of new technologies and technology-friendly public opinion in China. Exploring the perception and reception of anti-pandemic robots in different countries or cultures is important because it can shed some light on the future applications of robots, especially in the field of infectious disease control.

## 1. Introduction

Viruses, pandemics and the corresponding public health governance have been long-standing issues in human history. Setting up a new governance program often requires multiple validations to be progressively refined in the face of severe challenges from public health emergencies. The outbreak of COVID-19 has been a global stress test for both the health systems and the technical governance of every country [1]. In the early stages of the pandemic, robots, anti-pandemic apps and AI image recognition became essential intelligent anti-pandemic tools. They reflected an intelligent trend as a means to construct effective public health governance systems. This study is primarily concerned with public perceptions of robots used for medical care purposes in the pandemic; we refer to this class of robots as “anti-pandemic robots”, and we will further explain their features and functions in the first three parts of this paper.

In the fight against Ebola in 2015, the U.S. had already begun to recognize the potential of robots, suggesting clinical care, logistics and reconnaissance as the three significant areas for robotic applications. After the COVID-19 outbreak, this vision has quickly become a reality, with various types of intelligent robots taking on additional roles and functioning in several contexts throughout different stages of the pandemic’s development. At the start of the pandemic, most robots were not specifically developed for the unexpected outbreak of COVID-19, such as robots utilized to make deliveries. Many were not fully matched to the needs of the pandemic; subsequently, robots that better met the country’s anti-pandemic goals were devised as technicians raced against time. In China, they were quickly dispatched to hospitals, quarantine facilities and other places for emergencies in the early stages of the pandemic. However, once anti-pandemic behavior was common or the shortage of medical workers is alleviated, many such robots began to enter into daily life in large cities, such as temperature measuring robots in public areas like universities, banks and restaurants. Scholars from Pakistan, South Korea and Indonesia have focused on and recognize the effectiveness of anti-pandemic robots in China [2,3].

The impact of robots on specific medical practices, medical environments and even public spaces has gained the attention of the media and the population. While experts in robotics emphasize the importance of optimizing planning and investment in robotics research for better use in future outbreaks [4], this research is more concerned with the public’s perception and reception of these robots. The research on people’s attitude towards anti-pandemic robots’ application should ideally include three steps: before, during and after the COVID-19 outbreak. However, because of the urgency of the pandemic, we have discarded the “before” stage. When this urgency is alleviated, we consider it necessary to eventually explore the situation and learn from the experience to prepare the way for relevant research and applications in the future.

This study examined the perception and reception of anti-pandemic robots in China through a questionnaire survey. The survey included an examination of how respondents evaluated such robots’ functions, the principles and objectives that robots should be programmed to follow. Respondents were also asked about their attitudes towards robots and views towards the possibility of human laborers being replaced by robots. Given that robots may play an essential role in the prevention and control of infectious diseases in the future, it is essential to understand the public’s attitudes towards robots in order to maximize their effectiveness in future public health emergencies. It is hoped that the comparison of this study with other related studies will provide insights into the current and future applications of robotics and inspire further interdisciplinary and cross-cultural discussions. It should be noted that according to the questionnaire, 93.6% of respondents were pursuing or had completed a bachelor, master or even doctorate degree, which means that this paper mainly analyzes the perception and reception of anti-pandemic robots among the more educated population in China.

## 2. Literature Review

There have been some direct discussions about the use and impact of anti-pandemic robots during COVID-19 in the English, Chinese and Japanese academics and media. This paper focuses on four aspects, namely functional issues with anti-pandemic robots, issues concerning ethical principles of anti-pandemic robots, issues concerning the positioning of anti-pandemic robots and issues related to replacing humans with robots as detailed in the following sections.

### 2.1. Functional Issues with Anti-Pandemic Robots

Robots for infectious disease are used in four main scenarios, including clinical care, public safety; laboratory and supply chain automation; and out-of-hospital care, quality of life, and continuity of work and education [5]. Considering the possibility of the actual exposure of the people to anti-pandemic robots, the application of robots during COVID-19 can be divided into the following three types.

The first type is the robots directly used in medical settings and quarantine facilities. These robots are most widely applied to decontamination, body-temperature measurement, handling contaminated waste, contactless deliveries of food and medicine and work that involves a degree of danger and repetitiveness [4,6]. More technically demanding telemedicine services and surgical assistance roles are also crucial in ensuring uninterrupted medical services during a pandemic [7,8,9]. In addition to ordinary medical robots or robots in other fields for medical and quarantine facilities, several nasopharyngeal swab robots have been developed specifically for COVID-19 testing [10]. The pandemic has placed higher demands on the automation and safety of robots and has promoted the debugging and innovation of related robots in technology. For example, a medical robot arm that can be controlled by Bluetooth from a smartphone [11]; mobile robots using a visible light communication system, which reduces the influence of traditional radio systems on patients and medical equipment [12]; and the indoor positioning system of mobile robot using optical sensors, which greatly improves their accuracy and stability [13]. The situation further increases the possibility of robots being used for emergency response in the event of a public health emergency.

The second type is the robots indirectly fighting against the pandemic, such as the autonomous delivery robots (ADRs) that have previously been used outside hospitals and in quarantine camps [14] and Anki’s Vector companion robot designed to relieve loneliness for home users [15]. In Japan, robots that assist the elderly with conversation practice and cooking have also received much attention [16].

The third type is the robots equipped with expanded functions to address the secondary effects of the pandemic. For example, Scassellati and Vázquez [17] reported on the role of robots in areas such as education and the economy, and in China and Vietnam, robots have been used in the daily operations of hotels [18,19].

The first type is the most predominant type of “anti-pandemic robot” referred to in this paper, followed by the second type. In general, we cannot ignore the ethical issues arising from this process.

### 2.2. Issues concerning Ethical Principles of Anti-Pandemic Robots

The ethical issues arising from human–robot interactions in general have been inadequately addressed, and there have been few discussions so far of anti-pandemic robots due to their relative novelty. Nevertheless, since anti-pandemic robots are involved in both “roboethics” and “medical ethics” [20], we can consider the existing ethical concerns about safety, privacy and psycho-emotional aspects of healthcare robots.

The first concern is safety. Safety is the foundation of healthcare robotic applications and a precondition for human–robot coexistence. Although technological advances have improved its safety continuously, there are still many controversies over the applications of surgical robots [21] and healthcare robots, which might cause accidents. It is easy to identify the causes of the problems such as the functional defects, product quality, operation methods and application scenarios of robots. Still, it is challenging to locate the subject responsible for accidents as it involves ethical principles.

The second problem is privacy. In the process of medical care, a robot may record data of users, but the right of access to this information, its retention periods and record authorization all involve privacy risks [22]; however, these risks are arguably related to data-sharing issues and might be reduced by effective data-protection measures [23]. For example, “Health Information Technology for Economic and Clinical Health Act” (the USA, 2009), “General Data Protection Regulation” (EU, 2018), and “Data Security Law of the People’s Republic of China” (China, 2021) are the practices of using legal means to reduce the risk of privacy issues all over the world.

The third problem considers psychological and emotional issues. Sparrow R. and Sparrow L. [24] argued that robots could not meet the social and emotional needs of the elderly. Sharkey A. and Sharkey N. [22] summarize six possible ethical risks of nursing robots as follows: a potential reduction in the amount of human contact, an increase in the feelings of objectification and loss of control, a loss of privacy, a loss of personal privacy and personal liberty, deception and infantilization, and the circumstances in which elderly people should be allowed to control robots. Nevertheless, they emphasize that the elderly benefit from robot care and recommend taking effective measures to balance the care benefits and the ethical costs. Several recent studies have also validated that social robots can build close relationships with users and have beneficial effects on health-related well-being [25]. The outbreak of COVID-19 provides a new scenario for the discussion of this issue. Jecker [23] suggests that during a pandemic when social activities are constrained, the positive care environment created by social robots, even if not equivalent to interpersonal interactions, can be an effective way to alleviate the social isolation and loneliness of the elderly.

However, in some of the most recent studies on the reception of the application of anti-pandemic robots, there is little research exploring this topic. Instead, these studies focus more on functionality [2,26,27].

### 2.3. Issues concerning the Positioning of Anti-Pandemic Robots

Veruggio and Operto [28] summarize four different perspectives on how robots are perceived and positioned: robots are nothing but machines, robots have an ethical dimension; robots are artificial moral agents, and robots are the evolution of a new species. Shaw-Garrock [29] divides humanoid social robots into “utilitarian social robots” for instrumental or functional purposes and “affective social robots” that interact with humans on an emotional level and perhaps even companionship.

There are two central questions here: whether robots can be “ethical subjects” and whether we should give ethical meaning to robots [30,31,32]. However, there is no necessary logical relationship between the two, thus necessitating a multi-level view. The rights of robots have been the topic of futurists in the last century, such as the discussions in the article “Robots: machines or artificially created life?” [33] and the paper on “The legal rights of robots” [34], which have caused controversies in China since 2015. Some propose that “it is reasonable to give robots some rights” and “respecting robots means respecting human beings themselves” [35]. However, others insist that “robots are not human beings” and “robots cannot give rise to moral problems by themselves, nor can they make moral judgments” [36]. The controversy is whether robots should be regarded as mere “tools”.

### 2.4. Issues on the Replacement of Humans with Robots

In “The Second Machine Age” [37], the authors mention that robots and other digital technologies are acquiring skills and abilities at an extraordinary rate; therefore, there has never been a worse time for a worker with only ordinary skills and abilities. This means that it is essential to consider the potential ethical risks of robots’ applications. There are three main perspectives on the extensive use of anti-pandemic robots.

First, some people hold an optimistic view toward the prospect of anti-pandemic robots. People in the robot industry believe that the pandemic has driven a breakthrough in the systematic application of robots in medical systems and looking forward to the development of the robot industry [38,39].

Second, some people are concerned about the immediate and potential risks associated with replacing humans with robots. For instance, robots may take over the jobs of humans and threaten people’s livelihoods because they do not need face masks, healthcare or social distancing and do not strike for better conditions, and hence “entry-level, unskilled-labor jobs are going away because of robots” [40]. Furthermore, the application of anti-pandemic robots may accelerate the popularity of robots in other fields. As customers become more concerned about safety and health, the development of automation will be accelerated in catering, e-commerce, retail industries, education, etc. In the long term, some jobs might be performed by inexpensive robots [41]. A study conducted in Italy demonstrated that the increased robot use in factories did reduce the risk of workers contracting the virus during the COVID-19 pandemic, but researchers still believe that “its effects on employment rate and income risk bringing with them unintended and unpleasant re-distributional consequences that must be monitored and counterbalanced” [27].

Third, some scholars think that we should take a dialectical and concrete view towards the replacement of human workers with robots. Autor [42] argues that journalists and expert commentators overstate the extent to which robots will replace human labor and ignore the strong complementarities between automation and labor, productivity increases, rises in earnings and augmented demand for work. Garza and Zorthian [43] hold that although COVID-19 accelerates the process of robots replacing humans, the application of automation and artificial intelligence (AI) can free humans from dangerous or tedious tasks to undertake more intellectually stimulating ones. In Japan, the extensive use of robots can also alleviate the vacant position crisis [44]. Therefore, some scholars emphasize the need to pay attention to the two intrinsic dimensions of “robots working for people” and “robots taking over the importance of people” [20].

## 3. Hypothesis and Methodology

### 3.1. Hypothesis

As mentioned above, anti-pandemic robots have a wide range of applications, and their essential role has been recognized to varying degrees. Nevertheless, there are controversies about the ethical principles involved in human–robot interactions, the positioning of robots and the replacement of humans with robots. Thus, the application of anti-pandemic robots is a complex technical, social, psychological and ethical issue, and in-depth discussion is warranted on how the population defines robots and views the psychological and ethical issues arising from human–robot interactions. Compared with the exploration of attitudes towards robots in the West and in Japan, few explorations could be found in the academic community concerning China, and samples are limited but include Bartneck et al. [45], Evers et al. [46], Li et al. [47], Haring et al. [48], etc. Even more, there is very little relevant research focused on anti-pandemic robots. Given this, our paper focuses on Chinese people’s perception and reception of anti-pandemic robots and proposes the respective hypotheses to be validated in this study in light of the existing discussions.

The American philosopher of technology Carl Mitcham [49] suggests that the Chinese philosophy of technology community is closer to the “pro-technology” school of the American philosophy of technology and should “emphasize criticality”. In response, the Chinese scholar Wang Guoyu [50] argues that China’s “pro-technology” tendency has a “deep public opinion and cultural foundation”, and “the negative effects of technology that many developed countries faced are not encountered in the early years of China’s reform and opening up”. We speculate that this “pro-technology” tendency is also reflected in the Chinese public’s perception and reception of anti-pandemic robots, which can be elaborated in terms of the following six aspects.

During the pandemic, Chinese people were exposed to multi-functional anti-pandemic robots. Whether actively choosing or passively accepting robot services, practicality becomes an important indicator for people to measure the necessity of robot application, especially in assessing the matching degree between robot functions and personal needs and the experience of use that has a significant impact on people’s cognition. Facing the dangerous virus and the survival crisis, robots are sent to the frontline to undertake the necessary basic work. In the context of an urgent and exceptional pandemic, we speculate that the overall attitude of the public towards the application of anti-pandemic robots tends to be practical, and the evaluation of robots’ functions shows a pragmatic tendency.

**Hypothesis** **1** **(H1):** *Chinese people pay more attention to the practical functions of anti-pandemic robots*.

Due to the practicability of robots in pandemics, members of public may have reduced ethical requirements of them. Take privacy as an example. Existing research shows that Chinese people pay little attention to privacy. According to the survey results of “Potential Information Leakage Risk of AI” in the 2019 China AI Research Report, there was little difference between the percentage ofs respondents who were worried (36.7%) and those who were not (35.9%) [51]. Robin Li, CEO of the Chinese Internet company Baidu, once publicly stated that “Chinese people are willing to trade privacy for convenience in most cases” [52]. This feature may be reflected in their ethical views about anti-pandemic robots.

**Hypothesis** **2** **(H2):** *Chinese people pay little attention to the ethical issues such as privacy involved in anti-pandemic robots*.

Capurro [53] argues that people of different societies and cultures have different perceptions of robots. Lim et al. [54]’s summary of almost 20 years of empirical research related to human–robot interaction highlighted that culture and experience with robots influence people’s perceptions of social robots. The U.S. and Japan alone have received more attention due to the particularity of their attitudes towards it. Ramge [55] observed that “robots are enemies in Europe, servants in America, colleagues in China, and friends in Japan”. According to a small sample survey conducted by the University of Hertfordshire, U.K. respondents are very accepting of the use of computer technology in the home, but are less receptive to home robots. Nevertheless, they were open to them playing the role of assistants, servants or tools, but not friends or companions [56]. Findings like these indicate there are distinct cultural differences or identities with respect to attitudes to robots. There is not much discussion about robots within China. Given the expectations placed on robots during the pandemic, their role in combating pandemics may be an important factor influencing their positioning. In view of this, the perspective of Ramge [55] is informative and here we consider his view as a hypothesis.

**Hypothesis** **3** **(H3):** *Chinese people position anti-pandemic robots close to “colleagues”*.

The issue of humans being replaced by robots has raised strong vigilance in Europe and America, as mentioned earlier, while related discussion in China has just started. Chinese scholars seem to take this problem dialectically [57,58]. That is, to affirm the contribution and potential of robots in saving labor, there is no broad consensus in Chinese public opinion about the negative effects of the replacement of humans by robots at present. Moreover, the application of anti-pandemic robots is placed on the premise of reducing the risk of virus transmission and alleviating the shortage of manpower caused by unexpected outbreaks, a particularity that may further weaken people’s sense of crisis in the jobs of robot substitutes. Thus, we hypothesized:

**Hypothesis** **4** **(H4):** *Chinese people are generally optimistic about the issue of humans being replaced by robots*.

The above four hypotheses correspond directly to the content of our questionnaire. In addition, the following two associations are also worthy of attention: the association between “perception” and “reception and the association between respondent demographics and attitudes.

“Attitude” is divided into three components in social psychology: (1) a cognitive component, (2) an affective component and (3) action tendency cognition. All three influence each other [59]. There may be a correlation between the perception and reception of anti-pandemic robots among the Chinese people, and based on hypotheses 1 to 4 above; we speculate that this correlation is positive.

**Hypothesis** **5** **(H5):** *Chinese people’s current perception of anti-pandemic robots will promote their reception of robot applications*.

The Chinese people’s perception of AI shows certain demographic characteristics. According to CISTP [60], people aged 31–40 pay more attention to AI. Cmrc [61] shows a higher level of AI awareness among young people aged 18–30 and highly educated people with a bachelor’s degree or above, but there is little variation between regions. Robotics and AI are both high-level technologies, so we speculate that the demographics mentioned above may also be reflected in the perception and reception of anti-pandemic robots.

**Hypothesis** **6** **(H6):** *The demographics of respondents (age, education and occupation) may affect the perception and reception of anti-pandemic robots*.

**Hypothesis** **6-1** **(H6-1):** *The perception and reception of anti-pandemic robots of the elderly are more negative than those of other ages*.

**Hypothesis** **6-2** **(H6-2):** *The higher the education level, the more positive the perception and reception of anti-pandemic robots*.

Anti-pandemic robots are mainly used in hospitals and clinical isolation sites. Therefore, we speculate that the attitudes of medical workers in a co-operative working relationship with them may be different from other people. A Chinese AI researcher pointed out that AI did play an important role in the pandemic, but not a central one [62]. Most of the current robots perform a single function, especially those anti-pandemic robots utilized for emergent use. Sometimes issues such as technical difficulties, incompatibility between intended function and practical implementation, and regular maintenance occur in the use of the aforesaid robots. These issues may jeopardize the success of medical workers and thus affect their attitude. Moreover, the widespread application of anti-pandemic robots may result in medical workers losing their jobs, so such people may be more direct, more relevant and, theoretically speaking, pessimistic about the position of anti-pandemic robots and the issue of humans being replaced by robots. Thus, we hypothesized:

**Hypothesis** **6-3** **(H6-3):** *Medical workers have more negative perception and reception of anti-pandemic robots than others*.

Facing the questions of “What exactly is a robot?” and “What exactly can robots do?”, a Chinese engineer who takes part in design of anti-virus robots argues that one should not be misled by the Chinese translation of “robot”, i.e., “machine–human”, which is somehow like the German concept of “Maschinen-Mann” from the movie *Metropolis* (1927). In contrast, the English definition of “robot” has no human attribute. In fact, a robot as a concept is nothing more than an intelligent device. “Robot” in Chinese translation is a personalized expression that has affected the development of many robot products. The values of robots should return to their essence. Robots that can work in dirty, dull and dangerous environments are the most valuable ones according to Xu [63]. Thus, we speculate that technology R&D are positioning their robots to be more functional than other people.

**Hypothesis** **6-4** **(H6-4):** *Technology R&D workers position anti-pandemic robots closer to “tools”*.

### 3.2. Instrument

**Figure 1:**
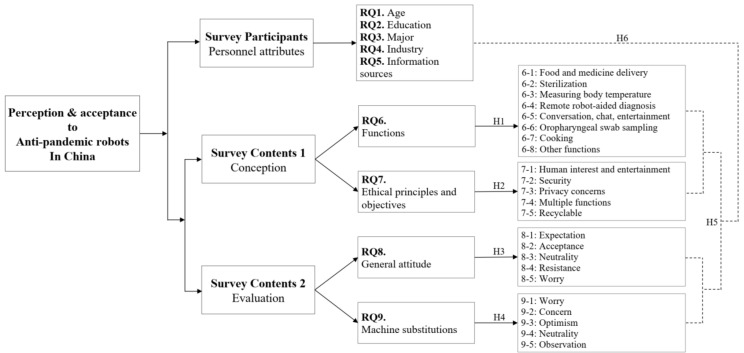
Research Framework.

The research framework of this study is shown in Figure 1. We investigated the perception and reception of anti-pandemic robots among the Chinese people, both in terms of the conception of the vision (including functional and ethical principles) and the evaluation of the reality (including positioning and social impact). To verify the hypothesis above, the data were collected by a survey with questionnaires in this study. The specific steps were as follows: First, we designed a questionnaire entitled “How do you think about the use of anti-pandemic robots during COVID-19?” (File S1). The “anti-pandemic robots” in this questionnaire referred to the physical robots that are used to fight against the pandemic in hospitals, quarantine facilities and some other contexts, excluding drones and outbound robots. The questionnaire consists of two parts. The first part is a survey of sample demographics, including four primary measurement indexes of age, education, major, occupation and information sources. The second part includes the functional evaluation of anti-pandemic robots in RQ6 (eight secondary indexes), ethical principles and objectives for anti-pandemic robots in RQ7 (five secondary indexes), general attitudes towards anti-pandemic robots’ applications in RQ8 (five secondary indexes) and views on the issue of humans being replaced by robots in RQ9 (five secondary indexes). A five-level scoring system was adopted from RQ6 to RQ9 (5-point scale, 1 = strongly disagree, 5 = strongly agree).

In the second step, a preparatory survey was organized for two medical workers, two technology R&D workers, two academic researchers of science and engineering and two academic researchers of liberal arts. The questionnaire was revised based on the responses and feedback from the respondents.

In the third step, the survey was conducted through the online survey platform “Wenjuanxing” (https://www.wjx.cn (accessed on 16 October 2021)) from November 14th to 27th, 2020. After the questionnaire was made public, the sample was collected by snowballing with the voluntary participation of the respondents, and a total of 1757 questionnaires were returned. After data cleaning, 1667 valid responses were retained (a valid return rate of 94.9%). The sample composition is shown in Table 1. The result distribution, mean (standard deviation) and the reliability and validity test results of RQ6-RQ9 are shown in Table 2.

The first half of 2020 is the most severe period of the pandemic in China when all types of anti-pandemic robots are most widely used and have the strongest presence in mass media. While in November, when we conducted this survey, the pandemic in China had gradually been controlled, and the urgency of the need for anti-pandemic robots had diminished, so did the attention of the media and the public. The results of the survey may vary from period to period. We chose this period because we believe that the attitude of the population is more stable at this time after the normalization of the pandemic.

## 4. Experimental Results

### 4.1. Functions of Anti-Pandemic Robots

Questionnaire RQ6 explored respondents’ evaluation of the importance of each function of anti-pandemic robots. The most highly recognized functions among all the options were the practical functions, such as sterilization (M = 4.52, SD = 0.84), temperature measurement (M = 4.27, SD = 1.01) and meal and medicine delivery (M = 4.23, SD = 1.06), followed by the more technically advanced functions of remote aid diagnosis (M = 3.70, SD = 1.18) and oropharyngeal swab sampling (M = 3.66, SD = 1.28). The additional functions were given relatively low importance such as chatting and entertainment (M = 3.14, SD = 1.24) and cooking (M = 2.99, SD = 1.24). This result verifies H1, that the Chinese people pay more attention to the practical functions of anti-pandemic robots.

The results of the cross-sectional analysis of the sample demographics showed that the effects of age and occupational background were relatively significant, and therefore, at this point, H6-2 is not valid (the relevant results of education and disciplinary background were omitted from Table 3). Among the four age groups, those under 40 years old attached more importance to the basic functions, while the middle-aged and elderly groups had relatively higher recognition of chatting and entertainment (*F*(3,1663) = 4.717, *p* < 0.01) and cooking functions (*F*(3,1663) = 6.230, *p* < 0.001). By the end of 2018, China had a population of about 167 million over the age of 65, including more than 40 million disabled senior citizens requiring about 20 million family service workers. Nonetheless, the actual supply in 2018 was only 3 million, leaving a huge gap [64]. Such a reality may cause the middle-aged and the elderly to turn to robots to meet their needs for life assistance as well as emotional communication. This result is a valid complement to H1. In addition, the young people did not demonstrate a cognitive advantage over the anti-pandemic robot function, except for the remote robot-aided diagnosis function (*F*(3,1663) = 4.595, *p* < 0.01), and therefore, H6-1 was not fully valid in the view about the function.

In terms of occupational background, medical workers overall had higher evaluations of the functions of anti-pandemic robots than other people; these included the additional functions, which were given less importance by others. This result differs from H6-3. This may be due to the fact that medical workers benefited directly from the application of anti-pandemic robots in the dangerous and laborious work of fighting against COVID-19. In addition, this survey finds that technology developers had the lowest evaluation of anti-pandemic robots, possibly due to an understanding of their functional limitations.

### 4.2. Principles and Objectives for Anti-Pandemic Robots

The results of RQ7 show that compared to “entertainment functions and human design” (M = 3.31, SD = 1.18), respondents were more interested in safety (M = 4.55, SD = 0.87), recyclability (M = 4.42, SD = 0.83), versatility (M = 4.38, SD = 0.86) and privacy (M = 4.35, SD = 0.84) of anti-pandemic robots; there was not much difference in the degree of importance among the latter, and therefore H2 is not valid.

In RQ7-3, which deals with privacy, we offered the seemingly unconventional perspective that “Any privacy concerns should be ruled out first, although anti-pandemic robots that not require much personal confidential data compared with retirement robots”. We know from technicians that anti-pandemic robots (such as the most widely used temperature measurement and sterilization robots) do not keep many sensitive personal data. This description was intended to prevent respondents from the misunderstanding that “all robots are closely associated with private data”, which may affect the objectivity of the results. Nevertheless, the results still clearly showed that respondents highly valued the possible privacy risks involved with anti-pandemic robots, and young people had significantly higher privacy awareness than the middle-aged and elderly (*F*(3,1663) = 6.388, *p* < 0.001). The results of this survey reflect a changing trend in the awareness of privacy.

In addition, the younger generation had a relatively higher demand for safety (*F*(3,1663) = 2.199, *p* < 0.10) and multifunctionality (*F*(3,1663) = 3.700, *p* < 0.05), showing a strong tendency toward “pragmatism”, while the middle-aged and elderly paid more attention to the entertainment function and human design (*F*(3,1663) = 3.360, *p* < 0.05). Some researchers have argued that it is unethical to use robots to solve the problem of elderly companionship and that we should respect the wishes of care recipients [24]. The results showed that the middle-aged and elderly in China did not express obvious resistance to robot-related functions but did hold certain expectations.

There were no significant differences in the requirements for privacy and recyclability among people with different occupations. Nonetheless, technology R&D workers, generally gave relatively lower evaluations, while medical workers had higher regard for entertainment functions and human design (*F*(3,1663) = 4.926, *p* < 0.01), and academic researchers showed greater demand for security (*F*(3,1663) = 4.654, *p* < 0.01). It was evident that the focus on the perception of this problem was different among medical workers, technology R&D workers, and academic researchers.

### 4.3. General Attitudes towards the Application of Anti-Pandemic Robots

According to the relevant discussions, we find three issues deserving our attention. The first issue is whether robots’ status can be further improved by their important roles in fighting against the pandemic. The second is whether human beings will continue to think about questions such as robots’ rights in the same way when facing dangerous and urgent situations themselves. The third is whether the difference in the views of philosophers and engineers indicates that people may have different perceptions due to different professional or occupational backgrounds.

Therefore, we designed five secondary indexes regarding the general attitudes towards anti-pandemic robot applications (RQ8): “Expectation: robots can be of great assistance in the future.” (M = 4.34, SD = 0.82); “Acceptance: intelligent robots demonstrate one positive aspect of advanced technology.” (M = 4.25, SD = 0.86); “Neutrality: robots are only technical tools.” (M = 2.92, SD = 1.28); “Resistance: I would rather be unattended than have a robot around.” (M = 2.01, SD = 1.16); and “Worry: robot applications have negative effects.” (M = 2.89, SD = 1.18). Overall, 84.9% and 81.1% of people tended to “agree” and “strongly agree”. The choice of “Neutrality” and “Worry” was polarized, but those who did not support these views were, respectively, 6.8 and 7 percentage points higher than those who did. Furthermore, 70.8% of people showed explicit “Resistance” (disagree + strongly disagree) on this question, indicating a clear tendency among respondents.

A comparison with the opinion of Ramge [55] mentioned in Section 3, “Hypothesis and Methodology”, reveals that a small number of respondents in this survey also agreed with the statement that “robots are tools” and with their negative impacts; however, people on the whole expressed dominant opinions that affirmed the empowerment of robot applications and supported technological development. People think that robots can be good “assistance” in the future but not simply a “technological tool”, which is clearly different from the positioning of robots as “enemies” and “servants”. However, given the question set in this survey, we cannot know exactly whether people will have friendly feelings towards robots. The emotional communication functions and human-interest design requirements of anti-pandemic robots for the elderly mentioned in Section 4.1 and Section 4.2 may suggest that we need to continue to pay attention to this issue. As far as the results of this survey are concerned, Chinese people position anti-pandemic robots closer to “colleagues” or “helpers”. Therefore, H3 is basically valid.

In this study, RQ8 and RQ9 were further used as target variables. The sample demographics and RQ6, RQ7, RQ8 (*Model 2*) and RQ9 (*Model 1*) were introduced as independent variables one-by-one into a hierarchical multiple regression analysis model (Table 4). This was intended to identify partial associations between the attitudes of the respondents and functional evaluations, design principles and attitudes towards the replacement of humans with robots.

Overall, there were no significant differences between the “expectation” and “acceptance” of future robot applications. However, the middle-aged and the elderly had relatively higher agreement with robot organon (*β* = 0.13, *p* < 0.001), “resistance” (*β* = 0.07, *p* < 0.01) and “worry” (*β* = 0.06, *p* < 0.05), so H6-1 is partially verified. In light of this, young people were more cautious about these problems. H6-1 is partially verified. Higher educated people have higher expectations about the future application of robots (*β* = 0.08, *p* < 0.01), so H6-2 partially holds. Among people with different occupations, the overall attitude of medical workers was the most positive compared to other groups who showed varying degrees of “concern” (*β*_T_ = 0.08, *p* < 0.01; *β*_A_ = 0.11, *p* < 0.01; *β*_O_ = 0.07, *p* < 0.10), so at this point, H6-3 is not valid. Among them, the technology R&D workers showed a stronger feature in the position of anti-pandemic robots, they were more inclined to consider robots as “tools” (*β* = 0.09, *p* < 0.01) and had the lowest favorability (*β* = 0.09, *p* < 0.01), so H6-4 was verified here. It can be seen that the views of Xu [63], the engineer mentioned above, are representative of the fact that, in the views of technology R&D workers, robots are only one of many technological tools, and they pay more attention to their practicality, while they are also better informed about the current state of robot functionality and may therefore position the human–robot relationship differently from the visionary hopes of other people.

The respondents’ positive evaluation of the functions and design principles of anti-pandemic robots clearly had an effective positive influence on their optimistic attitude. This significantly correlated to the recyclability (RQ7-5), safety (RQ7-2), entertainment function and humane design (RQ7-1), remote-aided diagnosis (RQ6-4), food and medicine delivery (RQ6-1) and sterilization function (RQ6-2) of the anti-pandemic robots. That is, H5 holds. It was also significantly correlated with the attitude towards the replacement of humans with robots.

### 4.4. Attitudes towards “Humans Being Replaced by Robots”

Based on the main ideas about robots replacing humans revealed in the literature review, the following five secondary indexes were set in RQ9: “Worry: I am worried because it may cause medical workers to lose their jobs” (M = 2.81, SD = 1.25); “Concern: I am concerned because the success of anti-pandemic robots will promote the use of robots in other industries” (M = 3.07, SD = 1.23); “Optimism: I am not worried because the robots undertake the dirty and tiring work, thus freeing medical workers to enable them to be engaged with more skilled work” (M = 3.66, SD = 1.14); “Neutrality: This problem should be treated critically. While these robots occupy some jobs, they will give rise to new jobs” (M = 4.04, SD = 0.95); “Observation: I’m not clear about this problem, and we have to wait and see” (M = 3.25, SD = 1.12). The results showed that the dialectical attitude received the most support (agree + strongly agree = 74.6%), followed by the “optimism” (58.0%) and “observation” attitudes (36.8%). Although a small number of respondents also held some concerns and worries, Chinese people tended to view the issue with a more dialectical and optimistic attitude than the pessimistic view presented by Hayasaki [40] and Thomas [41] in previous studies, so H4 basically holds.

Table 4 shows that young people appeared to be more anxious about this issue. In contrast to the middle-aged and the elderly, they needed to directly face the dilemma of robots replacing humans generated or being generated by the popularization of intelligent technology. It also found that people with lower degrees of education showed higher crisis awareness, which may be due to the fact that they are more likely to be replaced by robots in mechanical manual work and that it is difficult for them to find a new career through skill upgrading in a short period of time. The anxiety of these two groups revealed a strong sense of realism (H6-1 is not valid here). Respondents with higher education are inclined to be relatively optimistic on this issue (*β* = 0.08, *p* < 0.01), and here H6-2 partially holds. Notably, medical workers, who are considered most likely to be replaced by anti-pandemic robots, were the most optimistic among the occupational groups. H6-3 is again verified to be not valid here. As mentioned above, the medical workers gave positive comments as to the robots’ performance during the pandemic. Cheng L. [20] argues that we need to address the binary relations between the direct effects and potential threats of robots replacing humans when exploring this issue. The robots assisted medical workers in a highly sensitive emergency situation, significantly reducing the risk of them contracting the virus. Perhaps it can be appreciated that the importance of this relationship, which impacts the life and safety of workers, transcends concerns about the potential threat of “robots will take up some key positions of humans” [20]. The results of the survey of 41 medical workers in Colombia showed that, while respondents were positive in their attitude concerning the usefulness and benefits of robots during the pandemic, only 29.3% of respondents were explicitly unconcerned about whether robots would replace them. Moreover, they agreed that robots should perform only repetitive and uncritical tasks. The researchers believe that this “fear of replacement” in the medical community is worthy of attention [26]. In contrast, Chinese medical workers responding to this survey showed a more pronounced positive attitude towards the issue of collaboration or competition with robots.

By contrast, the evaluations on the functions of anti-pandemic robots (RQ6) did not have a significant impact on people’s attitudes towards anti-pandemic robots. This indicated that people’s worries about anti-pandemic robots did not focus on the dangerous medical positions in which anti-pandemic robots have exerted great influence, such as sterilization and temperature measurement, reflecting the respondents’ dialectical views on this issue. Of all the factors, people’s attitudes towards the replacement of humans with robots significantly correlated to their general attitudes toward anti-pandemic robot applications (RQ8). These two value judgments were mutually supportive, and the “expectation” and “acceptance” of anti-pandemic robots helped people to view this issue with a more optimistic attitude. Meanwhile, their “resistance” and “worry” were reflected in the corresponding negative attitudes; this showed that fears of robots replacing humans could not completely exclude the influence of personal and emotional factors.

## 5. Discussion and Conclusions

This study is the first empirical one to explore the Chinese public’s views on robots with a large sample size and to verify nine hypotheses accordingly. The results of the current survey demonstrate that Chinese people overall have a positive attitude toward anti-pandemic robots and agree that they play an effective role in reducing the burden of medical workers and the risk of virus transmission (H1 holds, H3 basically holds and H5 holds). Although a few respondents showed “worry” or “wait and see” attitudes towards the possibility of robots replacing humans, more than 70% tended to treat the problem dialectically, considering that “while these robots occupy a part of jobs, they will give rise to new jobs”, and nearly 60% were optimistic (H4 basically holds). In addition, differences can also be observed between demographics. Compared to the middle-aged and elderly, young people were generally more positive in their attitudes, more realistic in the functions they required of robots and in their views on roboethics, while showing a relatively stronger sense of anxiety regarding the issue of replacing humans with robots (H2 not hold; H6-1 partly holds). Higher education seems to indicate higher expectations for future applications of robotics, as well as relatively optimistic views on the issue of robots replacing humans, while respondents also showed no significant differences in their attitudes towards the functional and ethical requirements of anti-pandemic robots (H6-2 partly holds). Among different occupations, medical workers gave higher evaluations of anti-pandemic robots than others, and their recognition of the robots’ contribution to medical work in the present anti-pandemic seemed to outweigh medium- and long-term concerns about the potential that robots would become competitors in the future job market (H6-3 not hold; H6-4 partly holds). It can be seen that the Chinese public’s views on anti-pandemic robots are partially in line with our predictions, but there are also some new circumstances that differ from previous understandings, which we have analyzed as follows.

The formation of this optimistic attitude can be considered for two cultural reasons in addition to the recognized role of robots as anti-pandemic technology and the public’s affirmation of their functions. Firstly, Chinese people generally have a high acceptance of advanced technology, and robots are often regarded as a representative of it. According to the 2019 China Artificial Intelligence Research Report, 66.8% of respondents were optimistic about the prospects for the future development of AI [51]. The results of the survey AI in the Eyes of Ordinary People, conducted by Cmrc [61], also showed that 61% of the 3625 Chinese respondents expressed anticipation and 56% excitement. However, most U.S. respondents in the 2019 Edelman AI Survey expressed curiosity about AI (46%), followed by concern (33%) and optimism (32%) [65]. Furthermore, during the five years from 2012 to 2017, 80,000 respondents from 27 EU countries showed a markedly negative trend. Responses became more cautious towards the use of robots, and the author of the study argues that the reason for the growth of anxiety lies in the negative implication of machines replacing humans [66]. This kind of concern was also evident in surveys of the Swiss and French populations in 2007 [67] and in a survey of the U.S. population in 2015 [68]. In contrast, the Chinese public has a more pronouncedly positive bias in their attitude, including views on the issue of robots replacing humans, and this research on anti-pandemic robots shows a similar tendency. However, through the above studies, whether it can be assumed that a positive, optimistic and functional-oriented attitude towards robots has developed in China, we believe that further arguments and observations are needed.

A further reason for an optimistic attitude in Chinese society toward robot technology is the technology-friendly environment created by Chinese mass media. For instance, we searched the relevant news reports from January 1 to May 31, 2020, in Baidu, the largest search engine in China, with the keyword “anti-pandemic robot”, finding that out of 222 valid search results (excluding repeated publications of the same information on different platforms), only one message gave a negative impression of anti-pandemic robotics, which was largely due to the concerns about robots replacing humans. However, this singled-out negative message was actually a translation of an article by the foreign media [69], which does not mirror Chinese people’s attitudes. For the rest of the 221 reports, they are all positive or tend to be positive about various anti-pandemic robots. However, before the pandemic, there were already many discussions in the media in Europe and the U.S. concerning the issue of humans being replaced by robots. Gnambsa et al. [66] considered that the increased media attention and public discussion of robots in recent years might have shifted public opinions in a more critical direction. Others such as Manyika et al. [70] argued that by 2030 about a fifth of all jobs are projected to be replaced by robots.

The survey results show that the public’s attitude towards anti-pandemic robots is conducive to the popularization of robotic applications and even to the further construction of smart medical care in China. At the same time, we should not ignore an important issue: when faced with a survival crisis, the focus of public attention will shift more to the effectiveness of anti-pandemic means, which may lead to a decline in the importance of ethical issues such as privacy. In this case, does the optimism of the Chinese people against anti-pandemic robots imply a “function-oriented” value choice between functional benefits and ethical risks? We think it is still a question that deserves more exploration. The anti-pandemic robots have been used in the particular case of COVID-19, and their importance and utility will inevitably have influenced the ethical considerations of respondents.

The current study showed that people’s ethical requirements were increasing. For example, 81.4% of respondents believed that “privacy should be considered first” (see RQ7-3). However, these ethical needs had not become a “privacy anxiety” that would have a significant negative impact on people’s acceptance of robots. In regard to this, we have two speculations about this: first, some of the respondents were only conscious of the privacy risks associated with robots but did not generate concrete perceptions in real life; second, some of the respondents may be optimistic about technological developments and have a certain level of confidence that technological advances will solve privacy problems, which makes them view privacy issues based on a positive mindset rather than excessive anxiety. There has been little discussion about privacy in China in the past, but nowadays, more and more attention is paid to it in Chinese academia and online communities. There has also been a growing amount of legislation from government departments at all levels (e.g., the “Shenzhen Data Regulations 2021”) with increasingly specific provisions.

The results of this survey can offer reference information in the robot application in the field of public health in the future. For example, robots play a crucial role in some basic tasks such as disinfection, sterilization or temperature measurement, and their practicability is highly appreciated by the public. However, the public expects other functions, and the emphasis on ethics reflected in the survey results can provide a reference for the government’s policy formulation and technology R&D by enterprises and institutions. Thus, in the future, more attention needs to be paid to the ethical issues arising from the practical application of advanced technology, balancing ethical reflection with the application of technology.

The implications from this study are as follows: (1) future robot applications should pay more attention to the balance between functionality and privacy to meet people’s increasing awareness of privacy protection; (2) the needs of different groups of people should be taken into account (for example, anti-pandemic robots serving the elderly could focus more on living assistance, human–robot emotional communication and entertainment functions); (3) there is a need to plan ahead on the issue of robots replacing humans, without posing a significant threat to medical jobs, in order to facilitate optimal collaboration between medical workers and robots on specific clinical tasks; (4) the differences in perception and reception between different groups remind us that theoretical discussions, technology R&D and applications are rarely holistic, which leads to the ethical discussions not being fully functional. Under the premise of unity of purpose, the three areas should realize a closed loop in which academics, technologists and relevant practitioners promote each other’s integration by establishing a dialogue. For the application of intelligent robots in a public health emergency, the technical preparation and theoretical discussion needs to integrate with the feedback from the medical field, considering the specific problems in particular scenarios.

## 6. Plans for Future Research

Given that anti-pandemic robots may still play an important role in future pandemics, we believe that related studies should continue to deepen, and there remains room for improvement in the survey method and content of this study. Our ideas and plans for future research are as follows.

This survey was conducted with a sample of 1667 Chinese people, covering a certain range of ages, occupations and disciplines. A total of 93.6% of respondents were pursuing or had completed a bachelor’s, master’s or even doctorate degree. As the respondents in this survey were mainly highly educated people, we speculate that the main reason for this result is that robots are mainly used in medical facilities in large cities but less in remote areas. One of the prerequisites for respondents to answer this questionnaire was that they had had the opportunity to learn about robots, so residents of large cities with higher education levels dominated this questionnaire. In the data analysis, we examined 3 types of education, namely high school graduates, current/former bachelor’s or master’s degree students, and current/former Ph.D. candidates. We found that highly educated people seem to show a more optimistic attitude towards the application of anti-pandemic robots, but this difference is statistically weak; meanwhile, the large difference in the number of samples in these three categories will affect the objectivity of data analysis results. Therefore, we cannot make a definite judgment on the influence of educational backgrounds. However, we believe that the following hypothesis also deserves attention: people with a lower education level, and those living in remote areas, may have different attitudes towards robots than people with high education and living in large cities. At present, we have not yet obtained information on the use of anti-pandemic robots in rural China, but it is clearly of high academic value and practical reference significance to expand the sample size and increase its richness in future studies. At the same time, we also recognize the need to make relevant research more comprehensive by using other research methods, such as observations based on big data, interviews, case studies, etc.

Regarding the content of the questionnaire, we defined the concept of “anti-pandemic robots” at the beginning of the questionnaire and set four questions. However, considering that it may be burdensome for respondents if the questionnaire has too many questions, we chose to discard some detailed areas for the time being. For example, we did not further classify “anti-pandemic robots” except to give various functions. The survey of Colombian health care workers divides the anti-pandemic robots into threee types, telemedicine and telepresence robots (DIS), assistance and logistics robots (ASL), and telemedicine and telepresence robots (TEL)” [26]. The results showed that the respondents did have different functional expectations towards different types of robots. This is an important factor that should be considered in future research.

Meanwhile, countries have shown differences in anti-pandemic models and technical governance capacities at the beginning of the pandemic. The publication of “The Rome Declaration” (2021) and “Carbis Bay Declaration” (2021) reminds us of the importance and necessity of strengthening global public health governance collaboration when dealing with global public health emergencies. “The Rome Declaration” highlighted the need to strengthen the prevention and control mechanism through global cooperation in medical technology application and digital transformation of medical systems, which suggests the rapid adoption of intelligent technology in the global public health field and indicates that intelligent robots will continue to play an important role in fighting against infectious diseases. We have now learned to some extent about the application or reception of anti-pandemic robots in countries such as China, South Korea, Indonesia and Colombia. Still, more research should be done in the U.S., Europe and Japan, where the cultural characteristics of robots differ significantly. How does the population view the application of anti-pandemic robots? As mentioned earlier, although medical workers in Colombia and researchers in Italy were both positive about the usefulness of robots in reducing the risk of transmission during COVID-19 pandemic, they still showed negative views towards the issue of robots replacing humans [26,27], which indicates different characteristics from the Chinese population. However, another study with a small sample size found that American people’s acceptance of hotel robot service has increased significantly since the pandemic [71]. These phenomena remind us that it is worthwhile to explore to what extent the use of anti-pandemic robots during COVID-19 reflects the culture of robotics and even technology in different regions, or to what extent the viciousness of the pandemic caused the regions and cultures to discard their unique robotics cultures in the actual fight against the pandemic. We look forward to more related studies from other countries, since the similarities and differences in the attitudes of people reflected in these studies will be a direct inspiration for the application and placement of robots in the field of public health in different countries and even for related endeavors of international cooperation in the future.

What is more, the application of robotics has already had an impact on the overall environment of the medical facilities, and as a specific technological tool in this vastly integrated system, better planning and rational application can make it better match the overall medical environment and system. Therefore, people’s perception of the human–machine collaboration environment deserves attention in future research, and the results may in some way shed light on overall or partial environmental adjustments in hospitals so as to achieve better human–machine collaboration. Additionally, in a larger context, we can consider the following issues: in what way have external factors such as media reports, government policies, traditional culture and technical culture influenced the development of public health emergency response programs and the adoption of anti-pandemic robots in various countries? All these issues need further discussion. With similar studies conducted by scholars in other countries, we can develop a more comprehensive understanding of the issue through cross-sectional comparisons.

Anti-pandemic is a complex system that requires the cooperation of many parties and now seems to have evolved into a long fight. Through this survey, we have focused on the Chinese public’s views towards anti-pandemic robots, but this is only the first stage of our planned research, as the public’s attitude may be dynamic and new issues may arise from the progressively larger application of robots. There are scholars who have used historical cases of epidemics to point out that preparing in advance for future epidemic scan be costly, but fighting it without preparation can be more costly [72]. Therefore, the future will require more detailed, comprehensive and continuous observation, and we also hope to obtain useful inspiration from more related studies.

## Figures and Tables

**Table 1:** Details of the sample composition.

	Details
Sample Size	1667
Age	≤20 32.3%; 21–40 58.8%; 41–60 8.3%; ≥61 0.5%
Education	High school Graduates 6.4%; Current/former Bachelor’s or Master’s Degree Students 85.3%; Current/former Ph.D. Candidates 8.3%
Major	Liberal Arts 58.8%; Science and Engineering 29.2%; Liberal Arts & Science and Engineering 12.0%
Industry	Medical 12.1%; Technology R&D 7.3%; Academic Studies 32.0%; Other 48.7%
Information Sources	Real life 33.7%; News 71.7%; Japanese Anime 13.5%; Hollywood films 47.4%; Other (optional) 3.7%

**Table 2:** Descriptive Statistics of Variables of RQ6–RQ9.

	N	Mean	SD	α	KMO CVCR
RQ6 Functions of the anti-pandemic robot:					
6-1: Food and medicine delivery.	1667	4.23	1.06	0.78	0.80 57.30%
6-2: Sterilization.		4.52	0.84		
6-3: Measuring body temperature.		4.27	1.01		
6-4: Remote robot-aided diagnosis.		3.70	1.18		
6-5: Conversation, chat, entertainment.		3.14	1.24		
6-6: Oropharyngeal swab sampling.		3.66	1.28		
6-7: Cooking.		2.99	1.24		
6-8: Other functions.		3.19	1.12		
RQ7 Principles and objectives for the anti-pandemic robot:					
7-1: It should be of human interest and equipped with entertainment functions.	1667	3.31	1.18	0.73	0.74 50.17%
7-2: Robots’ security should be guaranteed first.		4.55	0.87		
7-3: Any privacy concerns should be ruled out first.		4.35	0.95		
7-4: Multiple functions should be developed to adapt to various situations in COVID-19.		4.38	0.86		
7-5: It should be recyclable and able to be used for other medical or nursing functions in the future.		4.42	0.83		
RQ8 Attitudes towards the application of the anti-pandemic robot:					
8-1: Expectation: robots will be of great assistance in the future.	1667	4.34	0.82	0.60	0.56 73.10%
8-2: Acceptance: intelligent robots demonstrate one positive aspect of advanced technology.		4.25	0.86		
8-3: Neutrality: robots are only technical tools.		2.92	1.28		
8-4: Resistance: I would rather be unattended than have a robot around.		2.02	1.16		
8-5: Worry: Robot applications have negative effects.		2.89	1.18		
RQ9 Attitudes towards the impact of the anti-pandemic robot:					
9-1: Worry: I am worried because it may cause medical workers to lose their jobs.	1667	2.81	1.25	0.56	0.57 67.53%
9-2: Concern: I am concerned because the success of anti-pandemic robots will promote the use of robots in other industries.		3.07	1.23		
9-3: Optimism: I am not worried because the robots undertake the dirty and tiring work, thus freeing medical workers to enable them to be engaged with more skilled work.		3.66	1.14		
9-4: Neutrality: This problem should be treated dialectically; while these robots occupy some jobs, they will give rise to new jobs.		4.04	0.95		
9-5: Observation: I am not clear about this problem, and we will have to wait and see.		3.25	1.12		

The values of RQ8-3, RQ8-4 and RQ8-5 were inverted for the analysis of reliability and validity. KMO: Kaiser Meyer Olkin; CVCR: Cumulative Variance Contribution Rate.

**Table 3:** Effects of age and occupational background in RQ6 and RQ7 findings.

	Age					Industry				
	≤20	21–40	41–60	≥61	*F*	Medical	Technology R&D	Academic Studies	Other	*F*
**RQ6-1**	4.17	4.26	4.24	4.56	*F*(3,1663) = 1.095, *n.s.*	4.35	4.01	4.24	4.24	*F*(3,1663) = 2.605, *p* < 0.05
**RQ6-2**	4.52	4.53	4.52	4.33	*F*(3,1663) = 0.161, *n.s.*	4.52	4.32	4.55	4.53	*F*(3,1663) = 2.559, *p* < 0.10
**RQ6-3**	4.31	4.25	4.22	4.11	*F*(3,1663) = 0.509, *n.s*.	4.27	4.09	4.29	4.28	*F*(3,1663) = 1.337, *n.s.*
**RQ6-4**	3.85	3.64	3.55	3.67	(3,1663) = 4.595, *p* < 0.01	3.60	3.55	3.70	3.74	*F*(3,1663) = 1.466, *n.s*.
**RQ6-5**	3.11	3.11	3.50	3.56	*F*(3,1663) = 4.717, *p* < 0.01	3.44	2.91	3.12	3.12	*F*(3,1663) = 5.450, *p* < 0.001
**RQ6-6**	3.66	3.64	3.78	3.56	*F*(3,1663) = 0.566, *n.s.*	3.72	3.58	3.66	3.65	*F*(3,1663) = 0.332, *n.s.*
**RQ6-7**	3.00	2.93	3.38	3.56	*F*(3,1663) = 6.230, *p* < 0.001	3.25	2.87	2.93	2.98	*F*(3,1663) = 3.886, *p* < 0.01
**RQ6-8**	3.30	3.12	3.27	3.67	*F*(3,1663) = 3.585, *p* < 0.05	3.29	3.08	3.17	3.20	*F*(3,1663) = 0.987, *n.s.*
**RQ7-1**	3.34	3.25	3.56	3.67	*F*(3,1663) = 3.360, *p* < 0.05	3.55	3.04	3.31	3.29	*F*(3,1663) = 4.926, *p* < 0.01
**RQ7-2**	4.60	4.55	4.40	4.33	*F*(3,1663) = 2.199, *p* < 0.10	4.49	4.31	4.63	4.55	*F*(3,1663) = 4.654, *p* < 0.01
**RQ7-3**	4.42	4.35	4.07	3.67	*F*(3,1663) = 6.388, *p* < 0.001	4.36	4.19	4.36	4.36	*F*(3,1663) = 1.174, *n.s.*
**RQ7-4**	4.48	4.33	4.34	4.22	*F*(3,1663) = 3.700, *p* < 0.05	4.39	4.20	4.42	4.37	*F*(3,1663) = 2.131, *p* < 0.10
**RQ7-5**	4.45	4.41	4.40	4.22	*F*(3,1663) = 0.597, *n.s.*	4.35	4.29	4.47	4.43	*F*(3,1663) = 2.001, *n.s.*

*n.s.*: non-significant.

**Table 4:** Intrinsic associations of respondents’ attitudes towards robots.

	*Model 1*					*Model 2*				
	RQ8-1	RQ8-2	RQ8-3	RQ8-4	RQ8-5	RQ9-1	RQ9-2	RQ9-3	RQ9-4	RQ9-5
	*β p*	*β p*	*β p*	*β p*	*β p*	*β p*	*β p*	*β p*	*β p*	*β p*
Step 1										
Age	−0.03	−0.04 ^†^	0.13 ***	0.07 **	0.06 *	−0.08 **	−0.08 **	0.07 **	−0.02	−0.08
Education	0.08 **	0.03	0.02	−0.04	−0.01	−0.06 *	−0.04	0.05 ^†^	0.05 *	−0.03
Major (Liberal arts = 0)										
Science and engineering (SE)	0.01	0.04	−0.01	−0.01	−0.06 *	−0.04	−0.03	−0.01	−0.03	−0.04
Liberal arts and SE	−0.02	0.04	−0.00	0.06 *	0.03	−0.02	−0.02	0.01	−0.02	−0.05 ^†^
Industry (Medical = 0)										
Technology R&D	−0.04	−0.04	0.09 **	0.09 **	0.08 **	0.01	0.04	0.01	−0.02	−0.02
Academic studies	−0.07	−0.05	0.02	0.06	0.11 **	0.14 **	0.12 **	−0.08 ^†^	−0.05	0.01
Other	−0.09 *	−0.04	0.08 *	0.05	0.07 ^†^	0.07	0.09 *	−0.08 ^†^	−0.06	0.01
*F*_1_	2.14 *	1.18	5.68 ***	2.98 **	3.18 **	6.36 ***	4.87 ***	3.53 **	.94	3.28 **
*R* ^2^_1_	0.01	0.00	0.02	0.01	0.01	0.02	0.02	0.01	0.00	0.01
Step 2										
RQ6 Functions										
RQ6-1	0.08 **	0.05 *	0.05 ^†^	−0.00	0.01	0.01	0.02	0.00	0.03	−0.02
RQ 6-2	0.05 *	0.04	−0.06 *	−0.09 **	−0.00	0.03	0.02	0.05 ^†^	0.04	0.02
RQ 6-3	0.05 *	0.04	0.04	−0.03	−0.03	−0.02	0.00	0.05 ^†^	−0.01	−0.00
RQ 6-4	0.10 ***	0.10 ***	0.01	0.00	−0.01	0.02	−0.02	−0.03	0.02	−0.05 ^†^
RQ 6-5	0.00	−0.01	0.02	0.03	0.00	0.01	0.06 *	0.03	0.05	0.03
RQ 6-6	0.04 ^†^	0.06 *	−0.02	−0.02	0.02	0.01	−0.00	0.01	0.01	0.00
RQ 6-7	−0.06 *	−0.03	0.02	0.04	−0.03	0.08 **	0.01	0.00	0.00	0.01
RQ 6-8	0.09 **	0.04	0.03	0.08 *	0.04	−0.00	0.01	0.02	−0.01	0.07 *
RQ7 Principles and objectives										
RQ 7-1	0.09 ***	0.12 ***	−0.05 ^†^	0.02	0.03	0.11 ***	0.08 **	−0.03	−0.06 *	0.05 ^†^
RQ 7-2	0.11 ***	0.08 ***	0.01	−0.01	0.01	0.02	0.02	−0.04	0.03	0.01
RQ 7-3	0.01	0.01	0.06 *	−0.02	0.02	0.06 *	0.05 *	0.02	0.03	0.06 *
RQ 7-4	0.01	0.04	0.02	0.04	0.04	−0.01	−0.01	0.07 *	0.01	0.01
RQ 7-5	0.18 ***	0.17 ***	−0.06 ^†^	−0.11 **	−0.04	−0.02	0.02	−0.00	0.08 *	0.08 *
RQ8 Attitude towards the application										
RQ 8-1	–	–	–	–	–	0.00	−0.01	0.13 ***	0.20 ***	−0.03
RQ 8-2	–	–	–	–	–	−0.05	0.02	0.18 ***	0.20 ***	0.02
RQ 8-3	–	–	–	–	–	−0.02	−0.00	0.12 ***	−0.00	0.07 **
RQ 8-4	–	–	–	–	–	0.22 ***	0.19 ***	0.06 *	0.02	0.12 ***
RQ 8-5	–	–	–	–	–	0.18 ***	0.20 ***	−0.07 *	0.03	0.17 ***
RQ9 Attitudes towards the impact				-					-	
RQ 9-1	−0.03	−0.06 *	0.06 ^†^	0.19 ***	0.15 ***	–	–	–	–	–
RQ 9-2	−0.00	0.02	0.09 **	0.15 ***	0.17 ***	–	–	–	–	–
RQ 9-3	0.09 ***	0.10 ***	0.16 ***	0.09 **	−0.02	–	–	–	–	–
RQ 9-4	0.23 ***	0.23 ***	−0.10 **	−0.06 *	−0.01	–	–	–	–	–
RQ 9-5	−0.06 **	−0.05 *	0.15 ***	0.14 ***	0.17 ***	–	–	–	–	–
*F*_2_	40.38 ***	36.73 ***	8.02 ***	16.31 ***	13.74 ***	16.51 ***	14.15 ***	13.04 ***	−20.11 ***	11.20 ***
*R* ^2^_2_	0.38	0.35	0.10	0.19	0.16	0.19	0.17	0.15	0.22	0.13
Δ*R*^2^_(1–2)_	0.37 ***	0.35 ***	0.09 ***	0.19 ***	0.16 ***	0.18 ***	0.16 ***	0.15 ***	0.23 ***	0.13 ***

*** *p* < 0.001, ** *p* < 0.01, * *p* < 0.05, ^†^ *p* < 0.10; – means that there are no corresponding results in this form.

## Data Availability

The datasets generated during and/or analyzed during the current study are not publicly available due to the funding principle but are available from the corresponding author on reasonable request.

## Supplementary Materials

The following are available online at https://www.mdpi.com/article/10.3390/ijerph182010908/s1, File S1: Questionnaire and basic findings of “What do you think about the use of anti-pandemic robots during COVID-19?”.
