# Supplementary material for: Public Perception and Reception of Robotic Applications in Public Health Emergencies Based on a Questionnaire Survey Conducted during COVID-19
Source: Int J Environ Res Public Health. 2021 Oct 17;18(20):10908. doi: 10.3390/ijerph182010908 (PMC8536172; doi:10.3390/ijerph182010908)
Supplement: Supplementary file 1 [file ijerph-18-10908-s001.zip › ijerph-1404404-supplementary.pdf]

File S1: Questionnaire and basic findings

What do you think about the use of anti-pandemic robots during COVID-19?

(English Version)

Statement:

1. The 'anti-pandemic robots' in this questionnaire referred to the physical robots that are used to fight against the pandemic in hospitals, quarantine facilities and some other contexts, excluding drones and outbound robots.
2. This questionnaire consists of 9 multiple-choice questions and is to be completed anonymously for the research activities of young researchers in universities only. All data will be kept strictly confidential.
3. Participants can obtain an analysis of the results of the questionnaire by leaving an email address.
4. To obtain more accurate results, we expect you to forward the questionnaire as well.
5. This questionnaire does not presuppose that you know everything about robotics, so you can choose your own opinion.

The COVID-19 outbreak in early 2020 has attracted a lot of attention with various robots taking on tasks such as sterilization, transport, temperature measurement, guiding, sampling, remote assistance and even cooking in isolated areas and hospitals. Your participation is important to better understand people's perceptions of the phenomenon of anti-epidemic robot use.

Thank you for your cooperation!

1. What is your age group? [Single choice] *

□ ≤ 20 32.3%

□ 21–40 58.8%

□ 41–60 8.3%

□ ≥ 61 0.5%

2. Your educational status is (including attendance)? [Single choice] *

□ Current/former bachelor's or master's degree student 85.3%

□ Current/former Ph.D. candidates 8.3%

□ Other 6.4%

3. What is your disciplinary background? [Single choice] *

□ Liberal arts 58.8%

□ Science and engineering 29.2%

□ liberal arts and science and engineering 12.0%

4. What is your industry? [Single choice] *

□ Medical 12.1%

□ Technology R&D 7.3%

□ Academic studies 32.0%

□ Other 48.7%

5. Where do you get your impressions of robots (not specifically "anti-pandemic robots", but robots in general)? [Multiple choice] *

□ Real life 33.7%

□ News 71.7%

□ Japanese Anime 13.5%

□ Hollywood films 47.4%

□ Other (optional) 3.7%

(The following questions are scored on a scale of 1 to 5, indicating increasing degrees)

6. How important do you think the following Functions of the anti-pandemic robots are? *

	1	2	3	4	5
1.Food and medicine delivery	3.2%	4.7%	14.2%	21.5%	56.4%
2. Sterilization	1.6%	1.9%	7.9%	20.0%	68.6%
3. Measuring body temperature	2.3%	4.1%	15.3%	21.1%	57.2%
4. Remote robot-aided diagnosis	5.0%	11.3%	25.1%	25.6%	32.9%
5. Conversation, chat, entertainment	10.3%	20.8%	31.7%	18.8%	18.5%
6. Oropharyngeal swab sampling	7.4%	13.0%	21.9%	22.0%	35.8%
7. Cooking	13.7%	21.1%	32.2%	18.5%	14.5%

8. Other functions	9.0%	12.7%	43.8%	19.0%	15.6%

7. What principles or objectives do you think should be followed for anti-pandemic robots? *

	1	2	3	4	5
1. It should be of human interest and equipped with entertainment functions.	8.3%	14.7%	33.6%	24.5%	18.9%
2. Robots' security should be guaranteed first.	1.5%	2.9%	7.4%	15.2%	72.9%
3. Any privacy concerns should be ruled out first although anti-pandemic robots do not require much personal confidential data compared with retirement robots.	1.5%	4.0%	13.1%	21.1%	60.3%
4. Multiple functions should be developed to adapt to various situations in COVID-19.	0.9%	2.8%	11.5%	27.4%	57.4%
5. It should be recyclable and able to be used for other medical or nursing functions in the future.	0.8%	2.0%	11.4%	25.9%	59.9%

8. What is your attitude towards the application of the anti-pandemic robots? *

	1	2	3	4	5
1. **Expectation**: robots will be of great assistance in the future.	0.6%	2.0%	12.5%	32.8%	52.1%
2. **Acceptance**: intelligent robots demonstrate one positive aspect of advanced technology.	0.8%	2.5%	15.6%	33.4%	47.7%
3. **Neutrality**: robots are only technical tools.	16.1%	21.9%	30.9%	15.8%	15.4%
4. **Resistance**: I would rather be unattended than have a robot around.	43.4%	27.4%	18.6%	4.6%	5.9%
5. **Worry**: Robot applications have negative effects.	14.8%	20.6%	36.3%	17.5%	10.9%

9. What is your attitude towards the impact of the anti-pandemic robots? *

	1	2	3	4	5
1. **Worry**: I am worried because it may cause medical workers to lose their jobs.	19.0%	21.5%	29.5%	19.1%	10.9%
2. **Concern**: I am concerned because the success of anti-pandemic robots will promote the use of robots in other industries.	13.0%	19.1%	30.8%	22.4%	14.6%
3. **Optimism**: I am not worried because the robots undertake the dirty and tiring work, thus freeing medical workers to enable them to be engaged with more skilled work.	5.0%	10.1%	26.9%	29.9%	28.1%
4. **Neutrality**: This problem should be treated dialectically; while these robots occupy some jobs, they will give rise to new jobs.	1.9%	4.3%	19.2%	37.2%	37.4%
5. **Observation**: I am not clear about this problem, and we will have to wait and see.	8.6%	11.2%	43.5%	20.6%	16.2%
